# Readiness to provide comprehensive emergency obstetric and neonatal care: a cross-sectional study in 30 health facilities in Tanzania

**DOI:** 10.1186/s12913-024-11317-0

**Published:** 2024-07-31

**Authors:** Damas Juma, Ketil Stordal, Benjamin Kamala, Dunstan R. Bishanga, Albino Kalolo, Robert Moshiro, Jan Terje Kvaløy, Rachel Manongi

**Affiliations:** 1 Kilimanjaro Christian Medical University College, Kilimanjaro, Tanzania; 2 Manyara Regional Secretariat, Manyara, Tanzania; 3 Department of Pediatric Research, Institute of Clinical Medicine, University of Oslo, Oslo, Norway; 4 Department of Research, Haydom Lutheran Hospital, Manyara, Tanzania; 5 Department of Epidemiology and Biostatistics, Muhimbili University of Health and Allied Sciences (MUHAS), Dar es Salaam, Tanzania; 6 Department of Public Health, St. Francis University College of Health and Allied Sciences, Ifakara, Tanzania; 7 Department of Paediatrics and Child Health, Muhimbili National Hospital, Dar es Salaam, Tanzania; 8 Department of Mathematics and Physics, University of Stavanger, Stavanger, Norway; 9 Department of Research, Stavanger University Hospital, Stavanger, Norway; 10 Ifakara Health Institute, Dar es Salaam, Tanzania

**Keywords:** Facility readiness, Quality of care, Obstetric, Neonatal care, Maternal care, SARA

## Abstract

**Background:**

Despite the global progress in bringing health services closer to the population, mothers and their newborns still receive substandard care leading to morbidity and mortality. Health facilities’ capacity to deliver the service is a prerequisite for quality health care. This study aimed to assess health facilities’ readiness to provide comprehensive emergency obstetric and newborn care (CEmONC), comprising of blood transfusion, caesarean section and basic services, and hence to inform improvement in the quality of care interventions in Tanzania.

**Methods:**

A cross-sectional assessment of 30 CEmONC health facilities implementing the Safer Births Bundle of Care package in five regions of Tanzania was carried out between December 2020 and January 2021. We adapted the World Health Organization’s Service Availability and Readiness Assessment tool to assess amenities, equipment, trained staff, guidelines, medicines, and diagnostic facilities. Composite readiness scores were calculated for each category and results were compared at the health facility level. For categorical variables, we tested for differences by Fisher’s exact test; for readiness scores, differences were tested by a linear mixed model analysis, taking into account dependencies within the regions. We used *p* < 0.05 as our level of significance.

**Results:**

The overall readiness to provide CEmONC was 69.0% and significantly higher for regional hospitals followed by district hospitals. Average readiness was 78.9% for basic amenities, 76.7% for medical equipment, 76.0% for diagnosis and treatment commodities, 63.6% for staffing and 50.0% for guidelines. There was a variation in the availability of items at the individual health facility level and across levels of facilities. We found a significant difference in the availability of basic amenities, equipment, staffing, and guidelines between regional, and district hospitals and health centres (*p* = 0.05). Regional hospitals had significantly higher scores of medical equipment than district hospitals and health centers (*p* = 0.02). There was no significant difference in the availability of commodities for diagnosis and treatment between different facility levels.

**Conclusion:**

Facilities’ readiness was inadequate and varied across different levels of the facility. There is room to improve the facilities’ readiness to deliver quality maternal and newborn care. The responsible authorities should take immediate actions to address the observed deficiencies while carefully choosing the most effective and feasible interventions and monitoring progress in readiness.

## Introduction

Maternal and newborn deaths are a global concern, therefore, there was a global consensus in 2015 on the need to reduce maternal mortality to 70 per 100,000 and neonatal mortality to 12 per 1000 live births by 2030 [1]. However, some countries have made little progress or stagnated in achieving the set goals; by 2020, about 287,000 women died globally due to pregnancy and birth-related causes, bringing the maternal mortality rate to about 223 per 100,000 live births [2].

Like many developing countries, Tanzania’s maternal mortality rate (104/100,000) is higher than the global mean target value despite a recent significant reduction from 454/100,000 in 2012 [1, 3]. From 2010 to 2022, Tanzania only managed to lower the neonatal mortality rate from 26 to 24/1000 [3, 4]. More efforts are, therefore, needed to attain the global target. Quality Comprehensive Emergency Obstetric and Newborn Care (CEmONC) is one of the recommended interventions to lower maternal and neonatal deaths and a target has been set that by 2025 at least 60% of women should be able to access emergency care within two hours of travel [5]. Nine cardinal items are expected to be offered in CEmONC centres: parenteral antibiotics, parenteral anticonvulsants, parenteral oxytocics, assisted delivery, manual removal of the placenta, removal of retained products of conception, basic neonatal resuscitation, cesarean section, and blood transfusion [6].

It is known that a majority of maternal and newborn deaths are preventable when quality healthcare is available [7]. Healthcare is deemed to be of good quality when it is effective, safe, people-centred, timely, equitable, integrated and efficient, and hence improving the health and well-being of individuals and populations [8]. It has been suggested that improving quality at the current level of healthcare access and utilisation will have significant benefits [9].

One prerequisite for good quality healthcare is facility readiness [10]. Health facility readiness is described by the World Health Organization (WHO) as its capacity to deliver the service it claims to be offering. Readiness includes the presence of trained staff, guidelines, infrastructure, medicines, and diagnostic tests [10–12].

Research in middle and low-income countries has consistently documented inadequate health facility readiness for the provision of maternal and neonatal healthcare although there are efforts to improve [9, 13, 14]. Although poor readiness was common, there were variations between countries, regions in the same countries, and the type of facility [9, 13, 15–17].

There is a paucity of peer-reviewed studies that have looked into readiness for CEmONC in Tanzania using the SARA tool [13, 18]. Nevertheless, studies have highlighted challenges and improvements in the provision of CEmONC in Tanzania including the views of healthcare workers and users [13, 18–20]. Studies in other middle and low-income countries have documented similar findings of inadequate staffing relative to the number of clients, inadequate supplies, equipment, and emergency transportation [21].

This study was conducted to assess the readiness of health facilities before the implementation of a 3-year continuous quality improvement (CQI) project, the “Safer Births Bundle of Care” (SBBC). The SBBC contains scientifically proven innovative clinical and training tools, combined with new strategies for establishing CQI efforts and sustaining improved care [22]. This study aimed to assess facility readiness to inform SBBC implementation and other stakeholders aiming to improve health facilities’ quality of care provision.

## Methods

### Study design and setting

This cross-sectional study was conducted in 30 health facilities in five selected regions of Tanzania: Manyara, Tabora, Geita, Shinyanga, and Mwanza [22]. These health facilities were selected based on the high burden of maternal and perinatal mortality and the volume of deliveries and aligned with the government’s strategic priorities. The selected regions account for about 25% of deliveries and about 35% of all maternal and newborn deaths in the country [22]. The study sites included four regional hospitals, 15 district hospitals, and 11 health centres.

The health delivery system in Tanzania is pyramidal, with dispensaries (small outpatient facilities) meant to serve a village, at the bottom. Then, there are inpatient health centres, followed by district hospitals (referral points for health centres). Higher up the pyramid we have regional, and then national hospitals. Some health centres and all hospitals are expected to provide CEmONC services. Usually, health centres would refer complicated cases, or when there is no appropriate medical personnel or supplies, to district hospitals which, in turn, might refer to regional hospitals.

### Data collection

Data collection was done using a questionnaire adapted from the WHO’s Service Availability and Readiness Assessment (SARA) tool that was initially developed with the participation of several partners to systematically gauge and monitor health services by generating tracer indicators related to the services including emergency obstetric care [10]. It was administered by trained research teams to facility in-charges and different health workers at each facility. Data collectors were trained for two days at the Haydom Research Centre on the data collection tools and data collection process. Data were collected using an electronic Open Data Kit and then transferred to Excel. Data were checked for correctness and appropriate remedial actions were taken when necessary. Data collection took place from December 2020 to January 2021.

### Variables

Our outcome variable was the availability of items for the provision of care for each domain in the facilities. We had 57 variables for five domains: seven for amenities, fifteen for equipment, eleven for staff, ten for diagnostic and treatment commodities, and fourteen for guidelines [10]. Next, we measured the average readiness score as an average of the readiness scores for amenities, basic equipment, commodities for diagnosis and treatment, staff, and guidelines. Explanatory variables were the health facility type, the frequency of facility governing committee meetings, and maternal and perinatal deaths surveillance and review (MPDSR) meetings.

### Data analysis

The data collected were entered into IBM SPSS Statistics version 20 software for analysis. For each item, a 0–1 item score was assigned, according to whether the item was in place or not at a facility. Then the percentage of facilities with the item in place was calculated. Readiness scores for each of the domains were calculated as the average of the percentage scores of the items in the domain. The overall readiness was calculated as the average of the scores for each domain, thus giving equal weight to each of the five domains. A comparison of item scores within domains across different levels of explanatory variables was conducted. Fisher’s exact test was used to test for any difference in item scores across different types of health facilities. For readiness scores, differences were tested by a linear mixed model analysis, taking into account dependencies within regions. Appropriateness of the model was checked by residual plots. We used *p* < 0.05 as our level of significance.

## Results

**Fig. 1:**
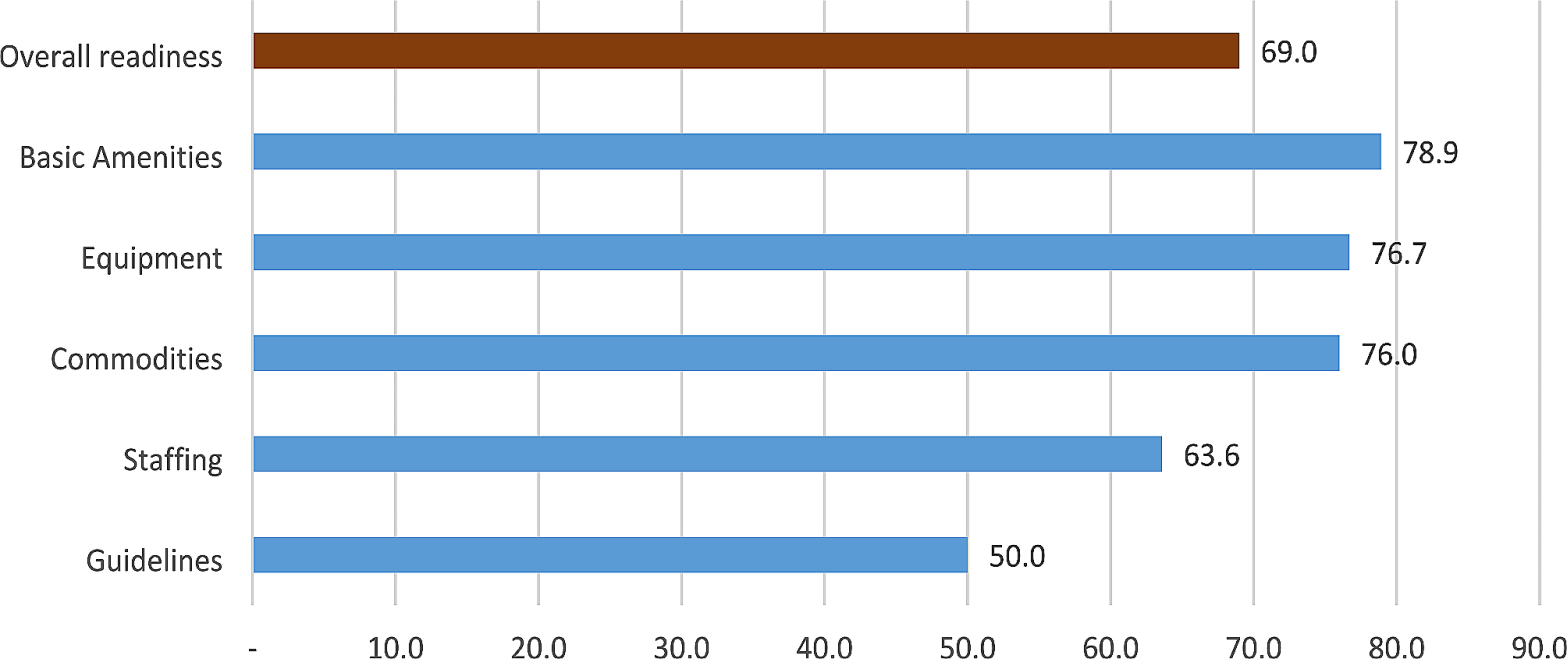
Facility readiness by domain in 30 health facilities providing CEmONC in five regions

A total of 30 health facilities were surveyed. Five readiness domains were assessed. The average scores were 78.9% for basic amenities, 76.7 for basic equipment 76.0% for commodities, 63.6% for staffing, and 50% for guidelines (Fig. 1). The overall readiness for provision of CEmONC services, including all domains, was 69.0%. Regional hospitals had the highest readiness score of 74.4% followed by district hospitals (71.4%) and health centers (64.8%). The difference in the overall readiness between the facility types was statistically significant by a linear mixed model (*p* = 0.014).

Table 1 shows the presence of amenities in the 30 health facilities surveyed. Most facilities had electricity from the national grid, solar power, or a generator. In the event of a power outage from the national grid, a backup source was available in 21 (70.0%) facilities. All facilities reported having potable water for facility use, but on the visit day, only 26 (86.7%) had running water.

90% of the facilities studied had a vehicle for emergency transportation. However, a third of ambulances in health centres had no fuel on the day we visited the facility. For all the items individually in the amenities domain there was no significant difference between facility types. For the average readiness score, we found significant differences across facility types, the lowest score being observed in the regional hospitals (*p* = 0.049).

**Table 1:** Basic amenities in 30 health facilities providing CEmONC in five regions

Items	Total	Regional Hospital	District Hospital	Health Centre	*P* value*
	*n* = 30	*n* = 4	*n* = 15	*n* = 11	
	n (%)	n (%)	n (%)	n (%)	
Grid electricity	29 (96.7)	4 (100.0)	15 (100)	10 (90.9)	0.50
Generator	20 (66.7)	1 (25.0)	11 (73.3)	8 (72.7)	0.21
Solar	10 (33.3)	0 (0)	4 (26.7)	6 (54.5)	0.13
Solar or Generator	21 (70.0)	1 (25.0)	11 (73.3)	9 (81.8)	0.15
Water on the visiting day	26 (86.7)	3 (75.0)	15 (100.0)	8 (72.7)	0.07
Emergency transportation	27 (90.0)	4 (100.0)	14 (93.3)	9 (81.8)	0.72
Fuel for ambulance	23/27 (85.2)	4 (100.0)	13/14 (92.9)	6/9 (66.7)	0.24
**Average readiness score**	**78.9**	**66.6**	**83.3**	**78.8**	0.049**

*Fisher’s exact test

**Linear mixed model

Table 2 shows the availability of equipment necessary to provide maternal and newborn care. On average, 76.7% of the facilities had the equipment needed. There was a variation in the presence of different equipment between facilities and facility levels. A caesarean section set, weighing scale, and penguin sucker were universally available. However, the presence of a wall clock in the room was in 22 (73.3%) facilities, a vacuum extractor was in 18 (60.0%), a small oxygen cylinder for transportation in 12 (40.0%), and a thermometer in 9 (30.0%) facilities. The difference in the availability of equipment between facility types was not significant except for newborn suction apparatus, which was less available in health centres and most available in district-level hospitals. The average readiness score for all the equipment was significantly different between health facility types (*p* = 0.020).

**Table 2:** Availability of basic equipment in 30 health facilities providing CEmONC in five regions

Items	Total (*n* = 30)	Regional Hospital (*n* = 4)	District Hospital (*n* = 15)	Health Centre (*n* = 11)	*P*-value*
	n (%)	n (%)	n (%)	n (%)	
Weighing scale	30 (100.0)	4 (100.0)	15 (100.0)	11 (100.0)	1.00
Thermometer (room)	9 (30.0)	2 (50.0)	6 (40.0)	1 (9.1)	0.18
Wall clock	22 (73.3)	3 (75.0)	9 (60.0)	10 (90.9)	0.19
Caesarian section set	30 (100.0)	4 (100.0)	15 (100.0)	11 (100.0)	1.00
Vacuum extractor	18 (60.0)	4 (100.0)	9 (60.0)	5 (45.5)	0.21
Newborn Resuscitation space next to delivery bed	18 (60.0)	2 (50.0)	9 (60.0)	7 (63.6)	1.00
Presence of newborn suction next to the delivery bed	25 (83.3)	3 (75.0)	15 (100.0)	7 (63.6)	0.02
Presence of bag/mask next to the delivery beds	24 (80.0)	3 (75.0)	12 (80.0)	9 (81.8)	1.00
Penguin sucker	30 (100.0)	4 (100.0)	15 (100.0)	11 (100.0)	1.00
Mask 0	29 (96.7)	4 (100.0)	15 (100.0)	10 (90.9)	0.50
Mask 1	29 (96.7)	4 (100.0)	15 (100.0)	10 (90.9)	0.50
Radian warmer	21 (70.0)	4 (100.0)	12 (80.0)	5 (45.5)	0.07
Oxygen cylinder/flow meter, humidifier	19 (63.3)	4 (100.0)	9 (60.0)	6 (54.5)	0.39
Small oxygen cylinder for transport (Availability)	12 (40.0)	2 (50.0)	4 (26.7)	6 (54.5)	0.35
Concentrator	29 (96.7)	4 (100.0)	15 (100.0)	10 (90.9)	0.50
Average score	**76.7**	**79.7**	**77.8**	**76.8**	0.020**

*Fisher’s exact test

**Linear mixed model

**Table 3:** Availability of commodities for diagnosis and treatment in 30 health facilities providing CEmONC in five regions

Items	Total (*N* = 30)	Regional Hospital (*N* = 4)	District Hospital (*N* = 15)	Health Centre (*N* = 11)	*P*-Value*
	n (%)	n (%)	n (%)	n (%)	
Haemoglobino-meter	18 (60.0)	2 (50.0)	9 (60.0)	7 (63.6)	1.00
Blood glucose	19 (63.3)	3 (75.0)	11 (73.3)	5 (45.5)	0.31
Dextrose 10%	25 (83.3)	4 (100.0)	13 (86.7)	8 (72.7)	0.52
Normal saline	29 (96.7)	4 (100.0)	15 (100.0)	10 (90.9)	0.50
Vitamin K	15 (50.0)	3 (75.0)	7 (46.7)	5 (45.5)	0.77
Ampicillin/gentamicin	20 (66.7)	3 (75.0)	11 (73.3)	6 (54.5)	0.57
Adrenaline injection	21 (70.0)	3 (75.0)	10 (66.7)	8 (72.7)	0.59
Phenobarbital injection	21 (70.0)	4 (100.0)	9 (60.0)	8 (72.7)	0.17
Magnesium Sulphate	30 (100.0)	4 (100.0)	15 (100.0)	11 (100.0)	1.00
Presence of safe blood all 90 days before the study	6 (20.0)	1 (25.0)	1 (6.7)	4 (36.4)	0.14
**Average score**	**(76.0)**	**(85.0)**	**(76.7)**	**(71.8)**	0.20**

*Fisher’s exact test

**Linear mixed model

On average, 76.0% of the facilities had the commodities needed for the diagnosis and care of mothers and children as shown in Table 3. About two-thirds of the facilities could test for blood sugar or haemoglobin, but less than half of the health centres could test for glucose compared to three-quarters of regional hospitals. The availability of magnesium sulphate (anticonvulsant) was universal, and that of normal saline was in 29 facilities (96.7%) as was that of 10% dextrose in 25 (83.3%). All health facilities could provide safe blood; however, only six facilities (20.0%) had blood on hand all the time in the 90 days before the study.

The presence of guidelines varied with the types of guidelines and facilities as presented in Table 4. Guidelines for postpartum haemorrhage were the most available in 28 facilities (93.3%) followed by those for pre-eclampsia and the poster for Help Babies Breathe (HBB). The newborn triage checklist was available in only four (13%) of the facilities. The availability of guidelines was the most prevalent in regional hospitals followed by district hospitals. The difference between facility levels was statistically significant (*p* = 0.03).

**Table 4:** Guidelines availability by type of facility for 30 health facilities providing CEmONC in five regions

	Items	Total *n* = 30	Regional Hospital *n* = 4	District Hospital *n* = 15	Health Centre *n* = 11	*p*-value*
	**Guidelines**	n (%)	n (%)	n (%)	n (%)	
1.	National neonatal guidelines	11 (36.7)	3 (75.0)	6 (40.0)	2 (18.2)	0.12
2.	Essential newborn care guideline	19 (63.3)	4 (100.0)	9 (60.0)	6 (54.5)	0.39
3.	Standard operating procedure set	8 (26.7)	2 (50.0)	3 (20.0)	3 (27.3)	0.55
4.	Newborn triage checklist	4 (13.3)	1 (25.0)	3 (20.0)	0 (0.0)	0.23
5.	Neonatal observation chart	12 (40.0)	4 (100.0)	7 (46.7)	1 (9.1)	0.00
6.	Discharge form	20 (66.7)	4 (100.0)	10 (66.7)	6 (54.5)	0.33
7.	Referral form	27 (90.0)	4 (100.0)	14 (93.3)	9 (81.8)	0.72
8.	HBB poster	24 (80.0)	4 (100.0)	12 (80.0)	8 (72.7)	0.83
9.	Prolonged labour	6 (20.0)	1 (25.0)	3 (20.0)	2 (18.8)	1.00
10.	Pre-eclampsia	27 (90.3)	4 (100.0)	14 (93.3)	9 (81.8)	0.72
11.	Antepartum haemorrhage	7 (23.3)	1 (25.0)	3 (20.0)	3(27.3)	1.0
12.	Postpartum haemorrhage	28 (93.3)	4 (100.0)	14 (93.3)	10 (90.9)	1.0
13.	Abnormal foetal heart rate (FHR)	5 (16.7)	1 (25.0)	3 (20.0)	1 (9.1)	0.66
14.	PPROM	12 (40.0)	1 (25.0)	9 (60.0)	2 (18.2)	0.09
	**Average Score**	**50.0**	**67.9**	**52.4**	**40.3**	0.03**

* Fisher’s Exact Test

**Linear mixed model

Key staff availability in the facilities studied is presented in Table 5 and shows variations between types of facilities and cadres. All health facilities had skilled nurses and medical officers were present in 28 (93.3%) facilities. The average readiness for staff was 63.6%. There were fewer staff in the lower health facilities and the difference was statistically significant (*p* = 0.009).

**Table 5:** Key staff availability by type of facility for 30 health facilities providing CEmONC in five regions

	Items	Total *n* = 30	Regional Hospital *n* = 4	District Hospital *n* = 15	Health Centre *n* = 11	*p*-value*
		n (%)	n (%)	n (%)	n (%)	
1.	**Paediatrician**	3 (10.0)	3 (75.0)	0 (0.0)	0 (0.0)	0.00
2.	**Gynaecologist**	6 (20.0)	4 (100.0)	2 (13.3)	0 (0.0)	0.00
3.	Medical doctor	28 (93.3)	4 (100.0)	15 (100)	9 (81.8)	0.24
4.	Assistant Medical Officer	22 (73.3)	2 (50.0)	12 (80.0)	8 (72.7)	0.55
5.	Clinical Officer	17 (56.7)	1 (25.0)	9 (60.0)	7 (63.6)	0.46
6.	Assistant Clinical Officer	7 (23.3)	0 (0.0)	5 (33.3)	2 (18.2)	0.59
7.	Nurse Officer	19 (63.3)	3 (75.0)	12 (80.0)	4 (36.4)	0.07
8.	Assistant Nurse Officer	30 (100.0)	4 (100.0)	15 (100.0)	11 (100.0)	1.00
9.	Enrolled Nurse	30 (100.0)	4 (100.0)	15 (100.0)	11 (100.0)	1.00
10.	Health information personnel	18 (60.0)	3 (75.0)	10 (66.7)	5 (45.5)	0.51
11.	Anaesthetists	30 (100.0)	4 (100.0)	15 (100.0)	11 (100.0)	0.13
	Average Score	63.6	72.7	66.7	56.2	0.009**

* Fisher’s exact test

**Linear mixed model

Table 6 shows key staff availability against the Tanzania staffing level recommendation. The availability of enrolled nurses (nurses with a basic certificate of training as opposed to more/longer trained nurse officers) was low in all levels of facilities. As per the guidelines, paediatricians and gynaecologists were available in referral hospitals, although a few district hospitals also had gynaecologists.

**Table 6:** Availability of key staff in different health facility levels in 30 health facilities providing CEmONC in five regions

SN	Cadre	Required per facility	Available in all facilities	Percentage Availability
**A**	**Health Centres (** ***n*** ** = 11)**			
1	Medical Officer	1	29	260.0
2	Assistant Medical Officer	1	16	145.5
3	Nurse Officer	0	9	NA
4	Assistant Nurse Officer	1	74	672.7
5	Enrolled Nurse	9	51	51.5
6	Anaesthetist	2	19	86.4
7	Gynaecologist	0	0	NA
8	Paediatrician	0	0	NA
**B**	**District Hospitals (** *n* ** = 15)**			
1	Medical Officer	8	92	76.3
2	Assistant Medical Officer	16	45	18.8
3	Nurse Officer	12	45	25.0
4	Assistant Nursing Officer	33	372	75.2
5	Enrolled Nurse	33	328	66.3
6	Anaesthetist	3	64	142.2
7	Gynaecologist	0	2	NA
8	Paediatrician	0	0	NA
**C**	**Regional Hospital (** ***n*** ** = 4)**			
1	Medical Officer	20	48	60.0
2	Assistant Medical Officer	23	11	12.0
3	Nurse Officer	31	9	7.3
4	Assistant Nursing Officer	77	117	38.0
5	Enrolled nurse	91	90	24.7
6	Anaesthetist	3	26	216.7
7	Gynaecologist	3	7	58.3
8	Paediatrician	2	5	62.5

The relationship between readiness and the frequency of the health facility’s governing committee and MPDSR meetings was also interrogated. Analysis of the data shows that all health facilities had facility governing committees. Most of the facilities (80.0%) had four meetings per year while two (6.7%) facilities met 12 times. Only one facility (a district hospital) reported no meetings in the year before the assessment. All facilities had MPDSR committees which met regularly. There was no significant relationship between the number of meetings conducted and the level of readiness of facilities.

## Discussion

This study assessed the readiness to provide emergency obstetric and newborn care services in 30 high-volume facilities across five regions of Tanzania. The overall readiness in the assessed facility was found to be 69% which is near 68% found a recent government of Tanzania assessment [23]. Our findings reveal readiness deficits across all domains, as documented by other studies, including the 2020 Tanzania Ministry of Health assessment report which involved 136 CEmONC facilities [18, 23, 24].

Nevertheless, the overall readiness was highest in regional hospitals, followed by district hospitals, and then health centres. This finding was consistent with other studies conducted in Tanzania and other low and middle income countries, where higher-level facilities demonstrate better overall readiness [18, 23, 25]. The finding also highlights a disparity: while CEmONC centres at any level are expected to provide similar essential services, mothers and newborns in lower-level facilities receive relatively poor service. In recent years Tanzania government has taken steps to improve existing facilities and construct new ones to allow surgical services, particularly caesarian section availability in health centres [23, 26]. This suggests that there is still unfinished work in capacitating health centres to meet the required readiness standards. Other factors, such as the low number of facility staff and their management skills, may also contribute to the lower readiness observed in these lower facilities [27, 28].

The facilities studied are meant to provide comprehensive emergency obstetric and neonatal care but improvement in several domains was needed to enable the service to be provided. These improvements have to be in a complete set in terms of human resources, equipment and amenities for the particular service. It might not be possible to provide quality care if one misses simple items like blood sugar measurement or basic antibiotics such as ampicillin and gentamicin. Other studies in Tanzania and other middle and lower income countries have documented similar inadequacies in meeting all requirements for the provision of cardinal functions for comprehensive emergency obstetric care [9, 13, 14, 16, 29, 30].

The findings, though, indicate areas where facilities are performing reasonably well like the general availability of water and electricity and the availability of key commodities such as magnesium sulfate and normal saline. This is in contrast to what was found in Ethiopia where only about a third of facilities had potable water [31]. However, facilities lacking running water on the day of the visit show that more efforts are needed to secure reliable water sources with possible backups or reservoirs in case of system breakdowns or shortages. This is important bearing in mind that water availability is important for almost all infection prevention activities and patients’ hygiene in the facility.

As documented by another study in Tanzania, electricity availability in assessed facilities was almost universal [18]. In Papua New Guinea, the availability of amenities including water and electricity was relatively poor, especially in lower-level health facilities, which differed from our findings [32]. Good availability of electricity in Tanzania facilities could be due to the efforts by the government of Tanzania to electrify every village and connect electricity to all government institutions in the villages [33]. It is, however, concerning to note that electricity backup was inadequate, particularly for higher-level facilities. This could be because backup for higher facilities needs large investments, be it for solar power or diesel generators, compared to lower facilities which need a small solar panel or generator affordable to the facility and easy to manage.

Most of health facilities had a vehicle for referral of patients, but some vehicles had no fuel ready, which was also a finding of the other studies conducted in Tanzania [30, 34]. This will inevitably cause delays in transporting patients, bearing in mind that only two thirds of Tanzanians are located within the two hour threshold to get to the referral point [35], and every second is important for saving lives. This is a complex issue as there are usually no petrol stations near health centres in rural areas of Tanzania making it difficult to refill timely [33]. Reasons for the absence of fuel when needed could come from inadequate financing, poor management of available fuel, including using the fuel and the vehicle for other administrative purposes, as documented by other researchers in Tanzania [34]. This calls for an immediate remedial action to rectify the situation looking at all layers of causes and relevant interventions.

Guidelines are a good tool to ensure that standardized evidence or consensus-based quality care is provided to patients [36]. The presence of guidelines varied with the type of guideline in question from 93.3% for postpartum haemorrhage management to 13.3% for the newborn triage checklist. This big range in the availability of different guidelines could be due to several reasons including service areas where government or implementing partners have given emphasis or prioritised training and distributing guidelines, and the time it has taken to penetrate the health system, as Pereira and others also documented [37]. For instance, in Tanzania, the newborn guidelines were completed in 2019 and had little time to penetrate [38].

However, it seems that guidelines are more readily available higher in the health system hierarchy. This may indicate a problem with ensuring that guidelines percolate down to the lowest healthcare delivery point required with accompanied dissemination. From experience, the few who have received training on the guidelines are usually asked to disseminate them to the lower level. Sometimes, there are not enough copies to distribute or no time and other resources to complete that task. A similar study in Tanzania using a national representative sample found that only 29.8% of facilities had appropriate guidelines [29]. In Nigeria and Brazil, guidelines were missing in all facilities studied for quality of care during childbirth [39, 40].

Guidelines are of value in ensuring the quality of health care, therefore, health managers need to devise innovative measures to make sure that guidelines are accessible and being used by practitioners. These measures may include leveraging modern digital technologies.

Our findings show that a majority of the facilities studied have the minimum number of skilled health workers to attend to maternal and newborn routine and emergency conditions. However, the nursing cadre, which is usually the first and critical in managing routine and emergency conditions, appears to be fewer in number than Tanzania staffing level recommendations. The lack of adequate staffing is ubiquitous in many African and other low and middle income countries [41]. Our study did not assess the quality of staff, though studies have shown that quality services may not be delivered as expected despite sufficient staff numbers [42, 43].

Previous research evidence suggests that there is a positive relationship between the presence of facility governing committees that discuss quality issues and quality improvement. In particular, quality improvement was more successful in facilities in which the health facility governing committees met at least monthly or weekly [44, 45]. In our study, all facilities had governing committees that met regularly but there was no association between meetings and readiness. This calls for more research to highlight the utility of these meetings and how to get the most out of them.

The strengths of this study include the fact that we visited the facilities and did not rely on self-assessment reports. The study also involved rural and urban facilities and different levels of the health system. However, there were limitations: we measured readiness but did not observe the services, and availability is not synonymous with use. Also, staffing was not adjusted to the number of clients or equipment in use. Another weakness is that the availability of commodities or amenities may vary over time while we only measured items on one visit.

## Conclusions

Despite a few areas with satisfactory availability of items, many areas still require significant improvement in amenities, equipment, commodities for diagnosis and treatment, staffing and guidelines. There is still a critical need to enhance health facilities’ readiness to deliver quality maternal and newborn care. The government, implementing and developing partners, and other stakeholders can achieved this by ensuring adequate amenities, equipment, commodities, staffing and guidelines while carefully choosing the most feasible and effective interventions. This will prevent unnecessary morbidity and mortality of mothers and their newborns. We strongly recommend a continuous quality improvement system to address these deficiencies. Additionally, regular assessments are crucial to monitor progress in facility readiness and ensure implementation of identified actions to improve quality of services.

## Data Availability

Data are available on reasonable request from Haydom Lutheran Hospital P. O. Box 9000 Haydom, Manyara, Tanzania Tel. +255(0)27 253 3194/5 Fax +255(0)27 253 3734, E mail: post@haydom.co.tz.

## Abbreviations

CEmONC: Comprehensive Emergency Obstetric and Neonatal Care
CQI: Continuous Quality Improvement
MPDSR: Maternal and perinatal deaths surveillance and review
PPROM: Preterm Premature Rupture of Membranes
